# Management of an Adult Open Diaphyseal Both-Bone Forearm Fracture With Severe Bone Loss: A Case Report, Literature Review, and Surgical Techniques

**DOI:** 10.7759/cureus.95733

**Published:** 2025-10-30

**Authors:** Bader Alotaibi, Raghad Alaujan, Husam Altahan, Abdullah M Alghamdi

**Affiliations:** 1 Department of Orthopedic Surgery, Ministry of National Guard Health Affairs, Riyadh, SAU; 2 Department of Orthopedics, King Abdulaziz Medical City, Riyadh, SAU; 3 College of Medicine, King Saud Bin Abdulaziz University for Health Sciences, Riyadh, SAU

**Keywords:** bone grafting, comminution, open forearm fracture, orif, trauma management

## Abstract

Open forearm fractures involving both the radius and ulna are challenging orthopedic injuries, typically resulting from high-energy impacts. These injuries require complex surgical interventions, such as open reduction and internal fixation (ORIF) or intramedullary nailing, and often necessitate multiple approaches due to the proximity of neurovascular structures. Severe cases can lead to complications such as infection, nonunion, and functional impairment of the limb. Here, we present the case of a 42-year-old male who sustained multiple injuries in a motor vehicle accident, including an open comminuted both-bone forearm fracture with severe bone loss. The fracture was initially managed at a local hospital with external fixation before the patient was transferred to a higher-level trauma center for definitive fixation. He subsequently underwent ORIF with bone grafting due to the extent of bone loss. This case highlights surgical techniques used in the management of open forearm fractures with severe comminution and bone loss.

## Introduction

Concurrent diaphyseal fractures of the radius and ulna are commonly referred to as both-bone forearm fractures. Open both-bone forearm fractures are characterized by bone comminution and significant bone loss, presenting a challenge in orthopedic trauma care [1]. These injuries often result from high-energy impacts, such as those sustained in vehicular accidents or severe falls, and require complex surgical interventions. Forearm fractures are common in emergency trauma settings, representing approximately 10% of all fractures. The distal forearm is the most frequently fractured site (47.8%), followed by the diaphysis of the distal third of the forearm (34.2%) [2]. Significant traumatic bone loss is associated with open fractures, reported in 11.4% to 40% of cases [3].

The unique anatomic and biomechanical complexities of the forearm, including the proximal and distal radioulnar joints and interosseous membrane, complicate surgical management and functional recovery, particularly in cases involving comminution and bone loss [4]. The proximity of neurovascular structures poses additional risks during reconstructive procedures and often necessitates a multidisciplinary approach [4].

Treatment strategies and surgical techniques discussed in the literature are primarily aimed at restoring alignment, length, and rotation of the forearm bones to regain functionality. Non-operative treatment is effective for pediatric forearm fractures and minor adult ulnar fractures. However, most adult forearm fractures require surgical intervention, typically through open reduction and internal fixation (ORIF) or intramedullary nailing. Hybrid fixation and specialized plating techniques may also be employed in specific fracture patterns to optimize healing and alignment [5,6].

Open fractures with extensive comminution and bone loss are less frequently reported and require tailored treatment approaches. Such injuries often lead to complications, including infection, nonunion, and functional impairment, posing additional challenges to standard orthopedic principles [7].

We present this case to describe complex surgical interventions for open comminuted forearm fractures with severe bone loss, incorporating a comprehensive review of both contemporary and traditional surgical techniques, along with an assessment of patient outcomes. We discuss initial management strategies such as infection prevention, followed by detailed surgical approaches including external fixation and intramedullary nailing, as well as advanced methods such as 3D printing and biologics for bone reconstruction. This case report highlights the importance of a multidisciplinary approach, emphasizing comprehensive rehabilitation to enhance both physical function and psychosocial recovery, and demonstrating the critical interplay between innovative surgical solutions and holistic patient care.

## Case presentation

**Figure 1:**
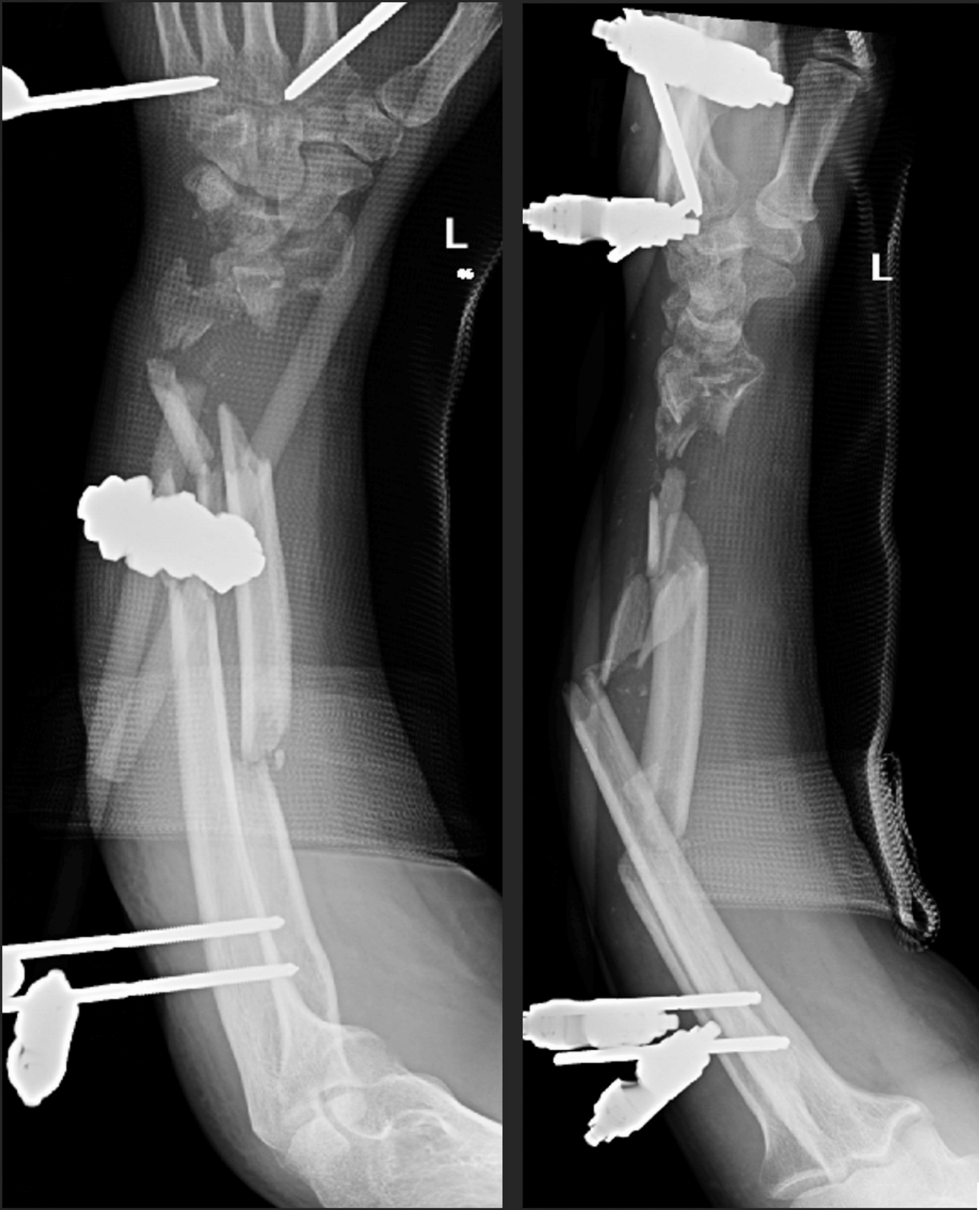
Anteroposterior (AP) and lateral radiographic views of the left forearm following Kirschner wire (K-wire) removal and application of external fixation.

A 42-year-old male with no relevant medical history was referred from a local hospital in Rafha, Saudi Arabia, following a motor vehicle accident. He presented with multiple injuries, including an open comminuted both-bone forearm fracture of the left upper limb. He had undergone preliminary K-wire fixation and skin grafting of the left forearm, along with repair of the extensor tendons in the left index, middle, and little fingers. Upon presentation to our hospital, the left upper limb, supported by a temporary splint, showed mid-forearm deformity and shortening with protective dressing, tenderness, and crepitus on manipulation, suggesting malalignment and instability. Detailed CT imaging of the left forearm revealed a severely comminuted fracture of the radius and ulna with malalignment. Following multidisciplinary case discussion and assessment, the patient was scheduled for a series of surgical interventions to address the complex injuries in the left forearm. The initial surgery involved wound irrigation and debridement of the left forearm. This required reopening the previously closed wounds, performing meticulous debridement and irrigation, re-closing the wounds, and applying a protective volar splint. The second surgery included removal of the preliminary K-wires and application of external fixation to the radius and ulna, followed by reapplication of a volar splint to maintain stability (Figure 1).

**Figure 2:**
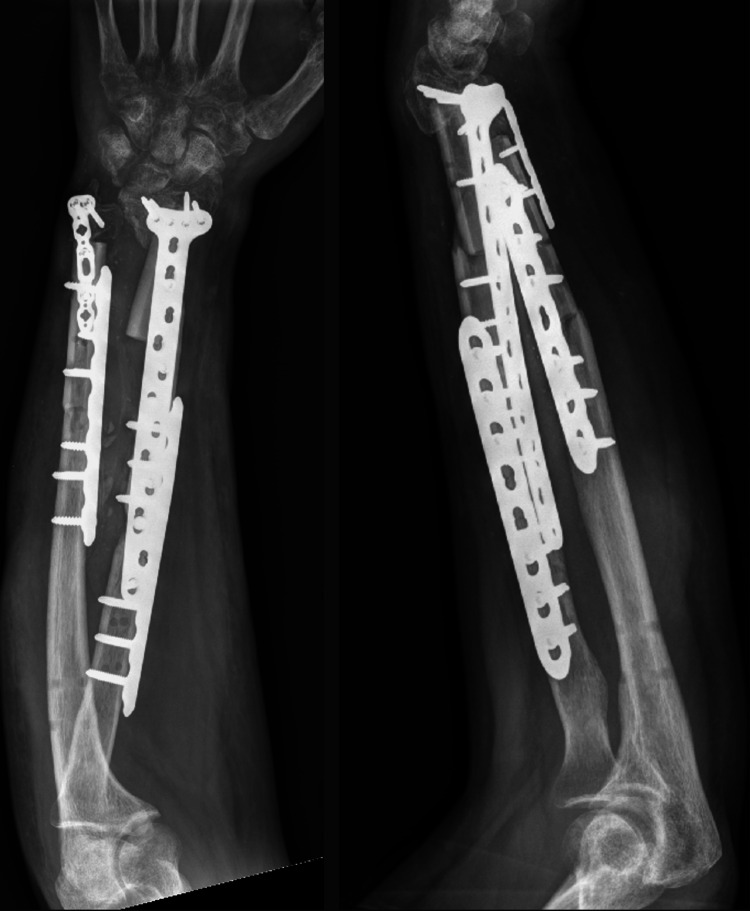
Anteroposterior (AP) and lateral radiographic views of the left forearm following open reduction and internal fixation (ORIF) with fibular allografts.

The final surgery consisted of ORIF with fibular allografts utilizing the volar Henry approach. To address the significant bone defect, approximately 4 to 5 cm in the distal shaft of the radius, fibular allografts were used, and fixation was achieved using a 12-hole two-column volar distal radius locking plate and an 8-hole lateral locking plate. The ulnar bone defect measured approximately 2 to 3 cm in the distal shaft of the ulna, which was managed with a fibular allograft and stabilized using a 2.4 mm L-shaped plate on the distal lateral side and a 7-hole locking plate on the volar side (Figure 2).

**Figure 3:**
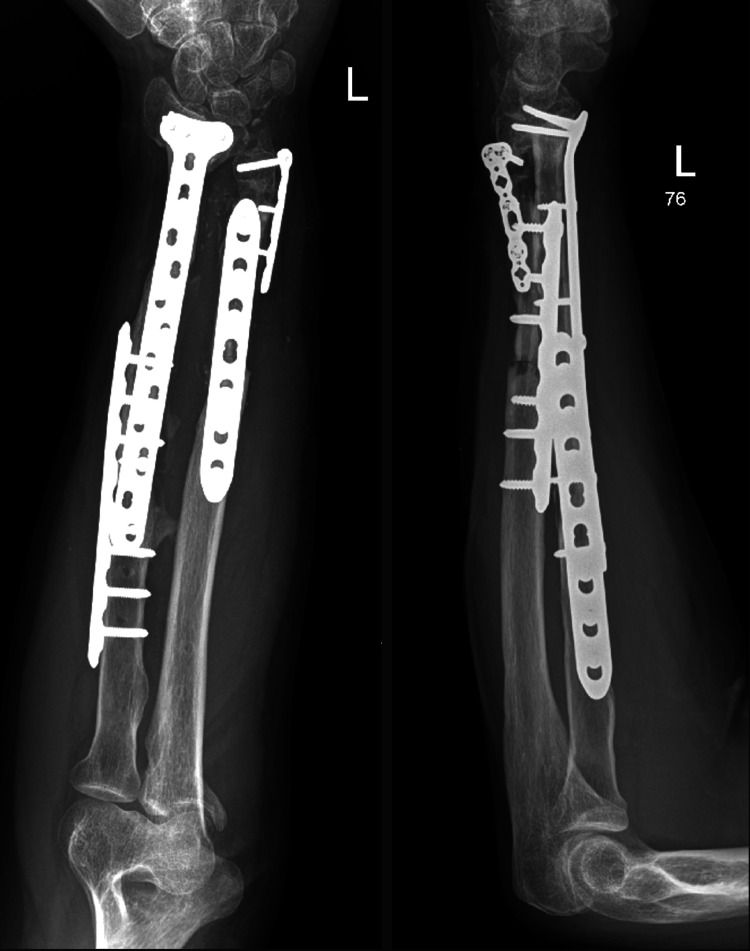
Anteroposterior (AP) and lateral radiographic views of the left forearm showing a healed fracture with a stable implant at the one-year follow-up.

**Figure 4:**
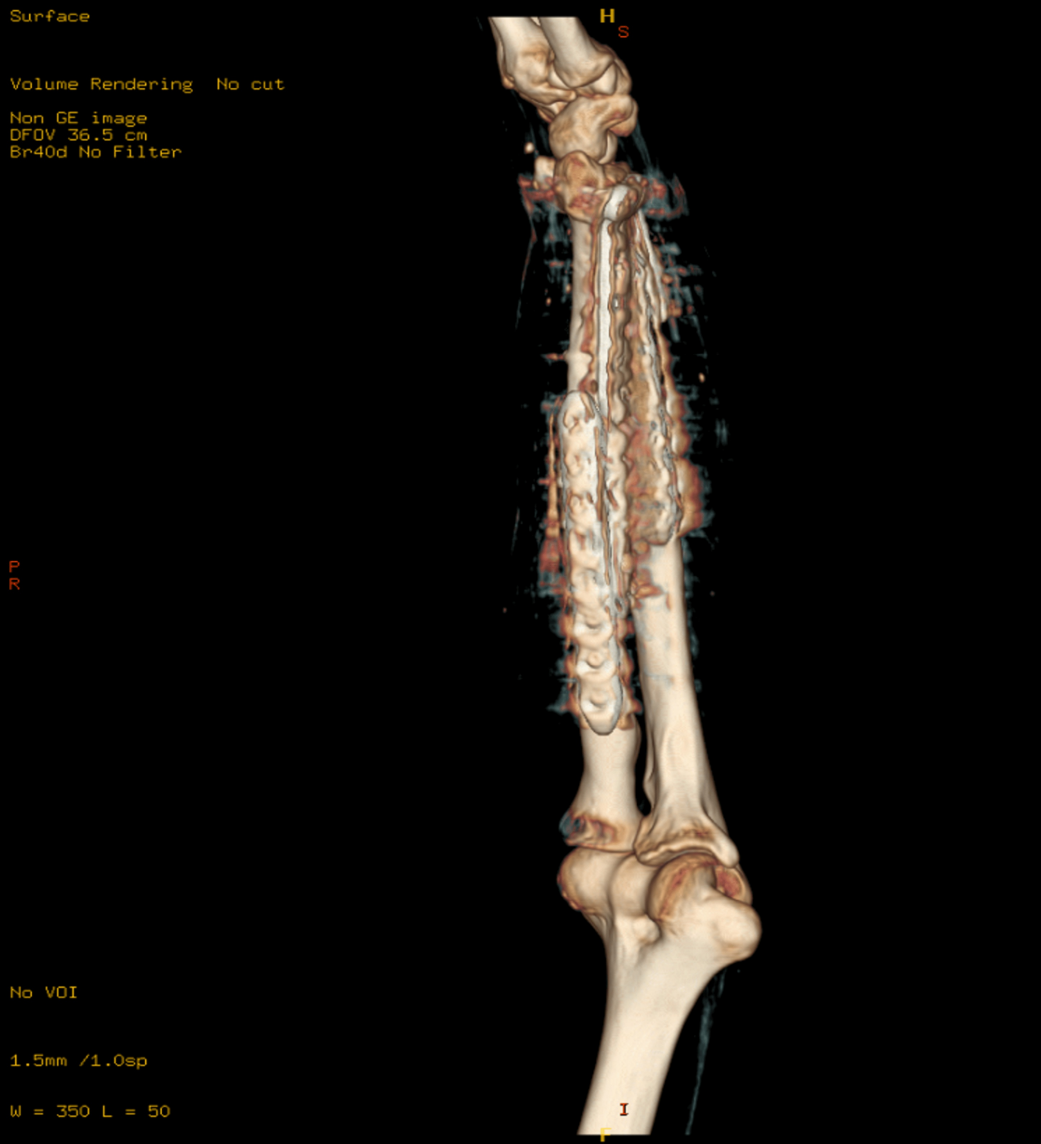
Computed tomography (CT) scan with three-dimensional (3D) reconstruction of the left forearm showing a healed fracture with a stable implant at the one-year follow-up.

**Figure 5:**
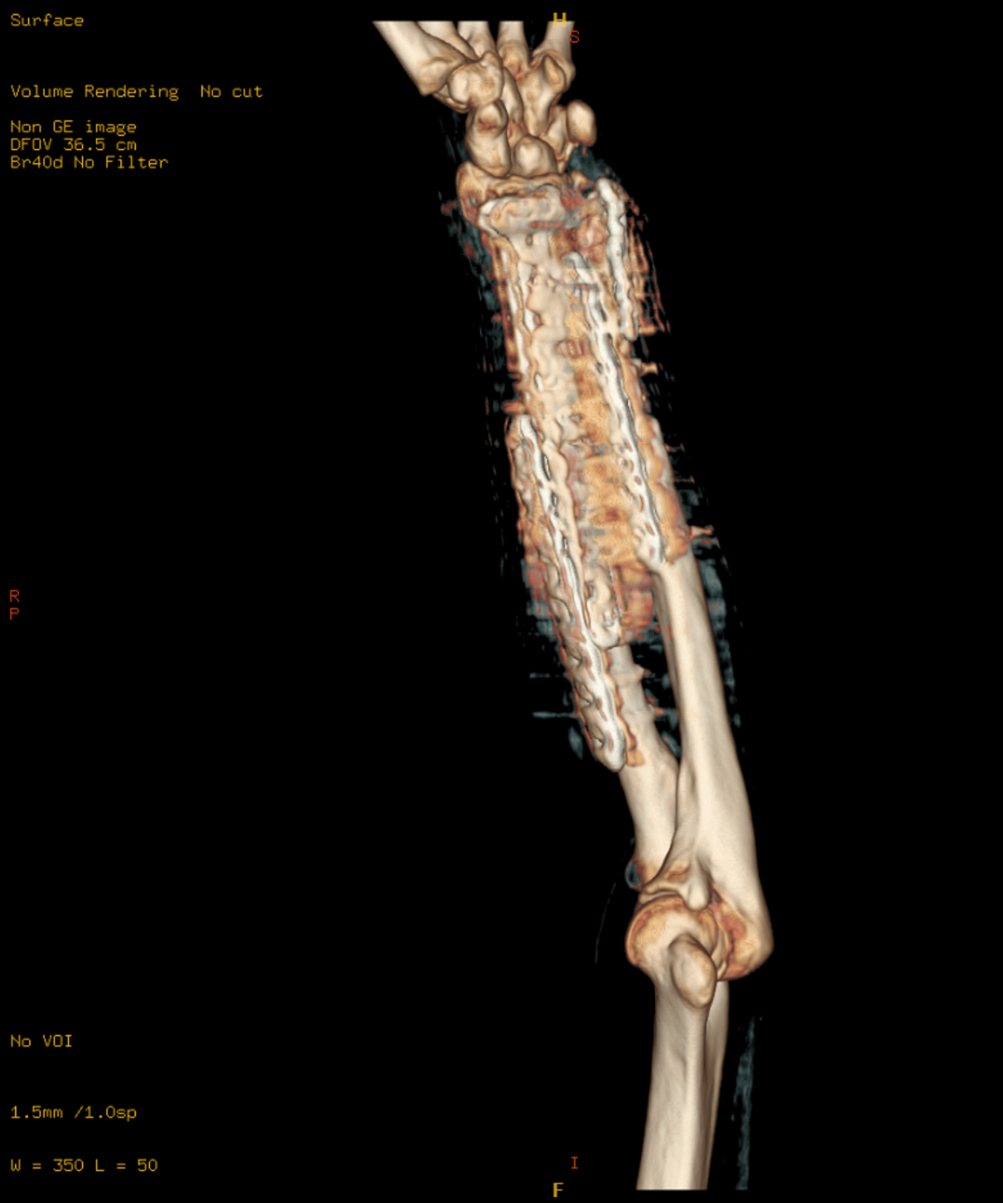
Computed tomography (CT) scan with three-dimensional (3D) reconstruction of the left forearm showing a healed fracture with a stable implant at the one-year follow-up.

**Figure 6:**
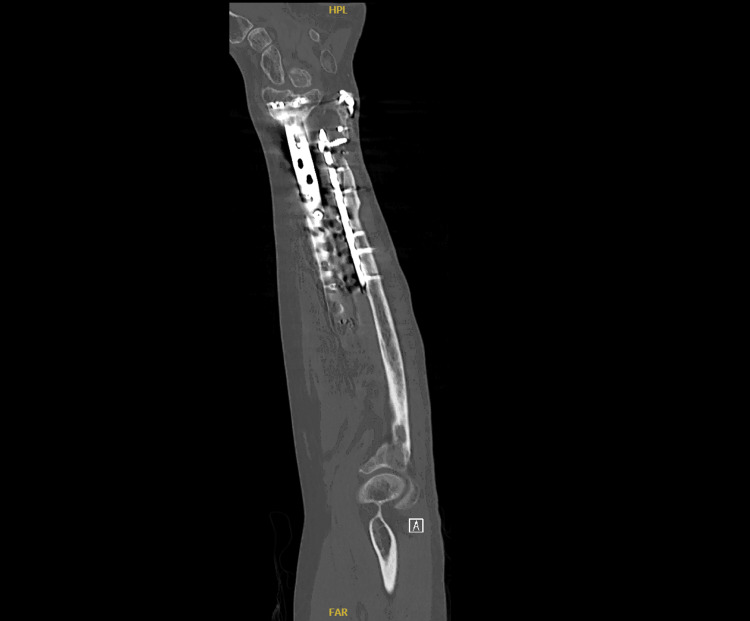
Computed tomography (CT) image of the left forearm showing a healed fracture with a stable implant at the one-year follow-up.

**Figure 7:**
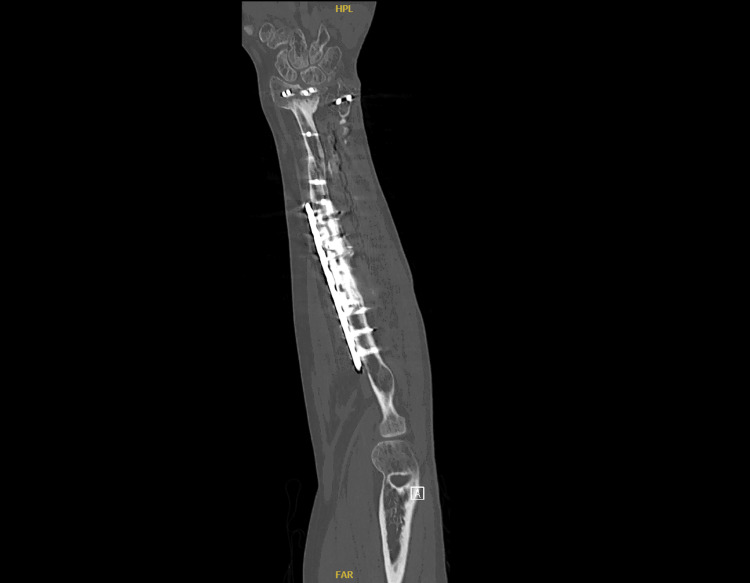
Computed tomography (CT) image of the left forearm showing a healed fracture with a stable implant at the one-year follow-up.

These surgeries collectively aimed to restore function, alleviate pain, and prevent long-term disability resulting from the severe fractures and soft tissue damage. The patient had an uneventful postoperative period and was discharged home. He was followed up in the outpatient clinic, initially two weeks after surgery to assess the surgical wounds, and subsequently at 4-6 weeks with X-ray imaging to evaluate fracture healing. Impressive bone healing and restoration of forearm function were observed. At the one-year follow-up, X-ray and CT images showed a stable implant with complete fracture healing and clinically improved outcomes (Figures 3-7).

## Discussion

Both-bone open forearm fractures with large bone loss are challenging orthopedic injuries commonly caused by high-energy impacts [8]. These injuries often require complex surgical interventions such as ORIF or intramedullary nailing. Severe cases may lead to complications including infection, nonunion, and functional impairment. This case demonstrates a complex injury managed through a series of surgical interventions, which resulted in improved functional outcomes and patient satisfaction.

Open fractures are typically managed by irrigation and debridement before definitive fixation to prevent infection [9]. The literature emphasizes the absence of infection after surgical debridement to highlight the importance of early and aggressive management of open fractures. A case series by Hurst MJ et al. demonstrated that early intervention with aggressive debridement significantly reduces the risk of osteomyelitis [10]. Debridement and the application of an external fixator are typically followed by definitive fixation with ORIF or intramedullary nailing; bone grafting is employed for bone loss in complex cases. This stepwise approach to open fracture management is supported by the literature, which underscores the role of external fixation as a temporary yet vital measure to stabilize complex fractures and allow soft-tissue healing before definitive surgery [11]. Nashi N and Kagda FH (2023) discussed concepts of bone grafting in trauma surgery, concluding that bridging large bone defects is essential to restore limb function [12]. The integration of fibular allografts and locking plates, as demonstrated in this case, has been associated with high rates of bone healing and satisfactory functional outcomes in the literature [13].

The recovery observed in this patient was marked by controlled pain and a gradual return to daily activities, providing a practical illustration of outcomes documented in similar trauma cases. The prognosis of open forearm fractures requiring extensive surgical intervention is generally unpredictable; positive outcomes are closely associated with prompt management, the extent of soft-tissue injury, and the precision of surgical technique. Similarly, Sop JL and Sop A (2017) demonstrated favorable outcomes in open fracture management when timely antibiotic administration (within 66 minutes) was combined with appropriate coverage for both gram-positive and gram-negative organisms, tetanus prophylaxis, and high-dose penicillin for clostridial contamination. These interventions, along with effective wound care and debridement, contribute significantly to the successful management of open fractures [14]. This case supports those findings, emphasizing the potential for favorable functional recovery even in the presence of severe initial injury. Furthermore, it reinforces the critical importance of postoperative care, including infection prevention through prophylactic antibiotics and regular monitoring, as evidenced by the absence of deep infection or osteomyelitis. This approach aligns with current best practices, which recommend antibiotic prophylaxis in the management of compound fractures [15]. Ultimately, this case highlights the need for a multidisciplinary approach involving orthopedic and plastic surgeons, as well as infectious disease specialists, to effectively manage complex forearm fractures. Individualized care strategies, including tailored surgical timing and fixation methods, are crucial for optimizing outcomes. The patient’s recovery illustrates the effectiveness of aggressive staged management for severe open fractures. Future directions should focus on refining proactive trauma care protocols, exploring innovative fixation techniques, and enhancing multidisciplinary collaboration to improve functional outcomes and promote recovery in complex orthopedic injuries (Table 1).

**Table 1:** Comparison of the present case findings with those reported in previous literature.

Aspect	Current Case Findings	Supporting Literature	Author (Year)
Cause of Injury	High-energy trauma causing an open comminuted both-bone forearm fracture	High-energy impacts are a common cause of open both-bone forearm fractures	Dankwa KA et al. (2024) [8]
Initial Management	Initial K-wire fixation, skin grafting, and tendon repair, followed by surgical debridement	Standard management begins with debridement and temporary stabilization	Srour M et al. (2015) [9]
Use of External Fixation	Applied after K-wire removal for temporary stabilization	External fixation is crucial for initial stability in severe open fractures	Liu et al. (2018) [11]
Definitive Fixation	Open reduction and internal fixation (ORIF) using volar plates and fibular allograft via the Henry approach	ORIF or intramedullary nailing are standard definitive fixation methods	Liu et al. (2018) [11]
Bone Grafting Technique	Fibular allografts used for radial and ulnar defects (4–5 cm and 2–3 cm, respectively)	Fibular grafts are effective in bridging large bone defects	Nashi N and Kagda FH (2023) [12]
Complication Management	No infection or osteomyelitis; staged surgery allowed optimal healing	Aggressive early management reduces infection risk	Hurst MJ et al. (2023) [10]
Functional Outcome	Impressive bone healing, restored function, and stable implant at 1-year follow-up	Timely staged procedures are associated with positive functional outcomes	Konstantinou P et al. (2024) [13]
Infection Prevention	Early and aggressive debridement with prophylactic antibiotics	Antibiotic prophylaxis is essential to prevent infection in open fractures	Appelbaum RD et al. (2024) [15]
Multidisciplinary Approach	Orthopedic and plastic surgery teams involved in case discussions and management	A multidisciplinary approach is vital for optimal outcomes in complex cases	Sop JL, Sop A (2017) [14]
Postoperative Follow-Up	Wound assessment at 2 weeks; imaging at 4–6 weeks and 1-year follow-up	Follow-up with clinical and radiological assessment is standard practice	Appelbaum RD et al. (2024) [15]

## Conclusions

Our case demonstrates the successful management of a complex open fracture involving both forearm bones with significant bone loss through a staged surgical approach. Initial debridement and external fixation, followed by definitive internal fixation with fibular allografts, resulted in excellent radiological and functional outcomes. The outcome of this case reinforces the importance of early intervention, meticulous surgical planning, infection prevention, and multidisciplinary collaboration in the successful management of high-impact open both-bone forearm fractures. Furthermore, it highlights the effectiveness of fibular allografting in bridging large bone defects and restoring forearm integrity in complex trauma cases, consistent with findings from previous literature.
